# Giant Condyloma Acuminatum of Vulva in an HIV-Infected Woman

**DOI:** 10.1155/2017/5161783

**Published:** 2017-04-13

**Authors:** Athanase Lilungulu, Bonaventura C. T. Mpondo, Abdallah Mlwati, Dismas Matovelo, Albert Kihunrwa, Balthazar Gumodoka

**Affiliations:** ^1^Department of Obstetrics and Gynaecology, University of Dodoma, College of Health Sciences, P.O. Box 395, Dodoma, Tanzania; ^2^Department of Internal Medicine, University of Dodoma, College of Health Sciences, P.O. Box 395, Dodoma, Tanzania; ^3^Department of Obstetrics & Gynaecology, Catholic University of Health & Allied Sciences, P.O. Box 1464, Mwanza, Tanzania

## Abstract

First described in 1925, giant condyloma acuminatum also known as Buschke-Löwenstein tumor (BLT) is a benign, slow-growing, locally destructive cauliflower-like lesion usually in the genital region. The disease is usually locally aggressive and destructive with a potential for malignant transformation. The causative organism is human papilloma virus. The most common risk factor is immunosuppression with HIV; however, any other cause of immunodeficiency can be a predisposing factor. We present a case of 33-year-old female patient, a known HIV patient on antiretroviral therapy for ten months. She presented with seven-month history of an abnormal growth in the genitalia that was progressive accompanied with foul smelling yellowish discharge and friable. Surgical excision was performed successfully. Pap smear of the excised tissue was negative. Despite being a rare condition, giant condyloma acuminatum is relatively common in HIV-infected patients.

## 1. Introduction

Anogenital warts, also known as condyloma acuminatum, are a common sexually transmitted disease among males and females caused by human papilloma virus (HPV) [1, 2]. The lifetime risk of acquiring HPV is between 50% and 80% [3]. It is estimated that among individuals who contact HPV, 90% will not develop genital warts, despite being able to transmit the disease [4]. Approximately 10% of individuals do not clear the infection and develop a persistent infection, with risk of developing genital warts [5]. Approximately 90% of genital wart cases are caused by low-risk HPV types (6 and 11) [1].

Giant condyloma acuminatum (GCA) is an extremely rare clinical form of genital warts, characterized by growth that extends into underlying dermal structures [6]. It is usually a benign, extensive cauliflower-like lesion commonly affecting the genitalia especially in immunosuppressed patients. Rarely, the lesion can extend to involve the anorectal region. GCA is usually nonresponsive to chemotherapy and radiotherapy. Local radical resection is mandatory for curative treatment [6].

We report a case of a 33-year-old HIV-infected patient on ART for ten months, who presented with seven-month history of progressive growth in the genitalia that was found to be GCA. Surgical resection was done successfully.

## 2. Case Presentation

We present a case of a 33-year-old female patient, Para 3+0, HIV-infected woman with baseline CD4-count of 30 cells/ul who has been on antiretroviral therapy (ART) for ten months prior to her visit. She presented to gynaecology clinic at Bugando Medical Centre, Mwanza, Tanzania, with seven-month history of progressive growths around genital region. The growths were of insidious onset, initially painless, although they later became painful. The lesion was initially noted on the edges of vaginal introitus; however they continued to spread to involve the perianal region. The growths were associated with foul smelling yellowish discharge. She has no history of receiving HPV vaccine.

**Figure 1:**
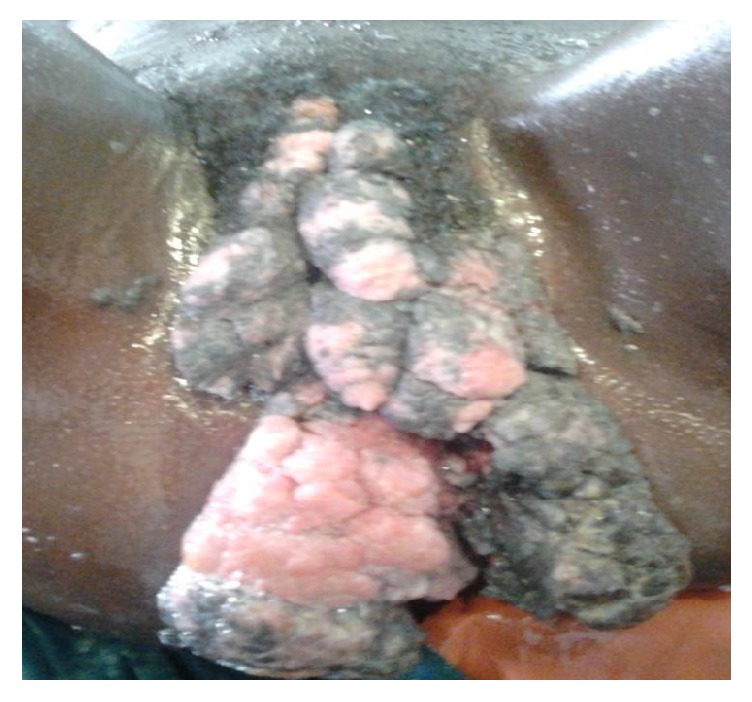
Giant condyloma acuminatum before surgical resection.

On examination, there were huge multiple masses, friable, cauliflower-like in appearance, dark grayish-pink in colour on her vulva involving both labia majora, extending anteriorly to the mons pubis and posteriorly to the perianal region, completely obliterating the urethral orifice, vaginal introitus, and anal opening (Figure 1). Masses were firm, edge growth, well-demarcated margins with rough surface. There were also some small satellite lesions on labia minora and the perianal region. There was also a foul smelling yellow vaginal discharge draining from multiple contoured margin areas on the masses.

**Figure 2:**
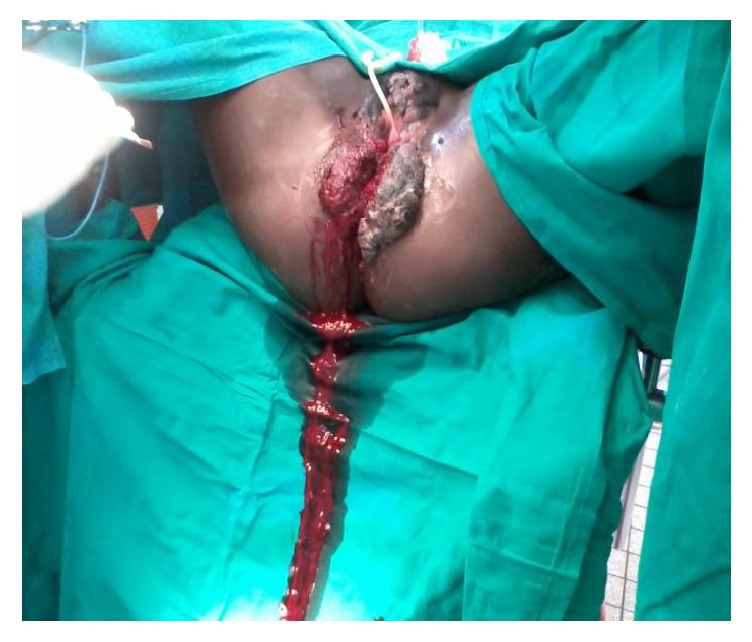
Giant condyloma acuminatum after surgical resection.

There was no peripheral lymph node enlargement. The vitals were blood pressure 110/65 mmHg, pulse rate of 78 beats per minute, respiratory rate of 20 cycles per minute, and temperature of 37°C. The diagnosis of GCA was reached. Due to the extensive nature of the lesions, surgical excision was planned and done successfully (Figure 2). The wounds healed by primary intention approximately one week after the procedure. Pap smear of the excised lesions was negative. Histological evaluation showed hyperkeratotic squamous epithelium underlying fibrous connective tissue stroma with nonspecific chronic inflammatory cells. There were no features of malignancy.

The patient was seen at one month and two months at which point she was doing fine, still on her ART with no recurrence of the vulval lesions. She was counseled on safe sex and other preventive measures for STIs. She was then lost to follow-up after six months of follow-up.

## 3. Discussion

Anogenital warts are the most common manifestation of genital HPV infection. Estimates show that among patients who contact HPV, only 10% develop genital warts [4], with HIV being the most significant predisposing risk factor. In rare occasions, anogenital warts may develop into extremely tumor masses, the so-called giant condyloma acuminata (GCA) or Buschke-Löwenstein tumors (BLT) [6]. These usually develop as cauliflower-like masses in the vulva although in rare cases like in our patient may extend to involve the perianal region. The tumor is slow-growing, highly destructive to contiguous tissue, and on rare occasions, it can metastasize. It is considered to be a regional variant of verrucous carcinoma [7].

GCA is usually a benign lesion with 30–56% chance of malignant transformation [8], being more common in men with a male to female ratio of 2.7–3.5 : 1. It is more common in men who are uncircumcised. The most common site for this tumor is on the glans penis; however, it can also be found on any anogenital mucosal surface like vulva, vagina, and rectum. Histologically, GCA exhibits features of pseudoepitheliomatous proliferation and local invasion by massive epidermal hyperplasia, hyperkeratosis, and parakeratosis; it is also markedly exophytic [9].

GCA is a rare tumor. It is even rare when in regions other than the penis like in our patient. Estimates show that GCA accounts for 5–24% of the penile cancers. The tumor has been shown to be associated with HPV subtypes 6 and 11 [10], the low-risk subtypes. Occasionally, however, it can be associated with the high risk subtypes (16 and 18). In one occasion the tumor was found to be associated with HPV subtype 54.

GCA usually starts to grow as a keratotic plaque that expands slowly into a cauliflower-like mass. The growth takes longer in immunocompetent individuals. In immunosuppressed individuals, however, the growth is commonly more rapid [11]. The lesion may then ulcerate; typically the lesion may be associated with foul odor like in our case. Regional lymph node enlargement is common usually following secondary infection; our patient, however, did not have enlarged lymph nodes.

The treatment of choice for the management of GCA is considered wide surgical excision [6, 12]. Surgical excision alone has been shown to result into disease-free state in up to 46% of cases [13]. Oral and topical chemotherapeutic modalities can be used as adjuvant, to surgery. Some factors that need to be taken into account during treatment choice include the size and thickness of the lesions, anatomic site, associated HPV subtype, and the immune status. For giant lesions like in our patient, topical therapy alone is generally insufficient to control disease. No medication, however, can eradicate HPV infection. The role of radiation therapy remains controversial. The role of ART is not clearly defined. This patient had been on ART for about ten months without any improvement on her lesions. Other reports also show that the use of ART does not improve HPV-associated lesions [14].

## 4. Conclusion 

Despite being a very rare manifestation, giant condyloma acuminatum of the vulva occurs, especially in immunosuppressed patients. Surgical excision remains the mainstay of treatment with adequate cure rate and limited recurrences. HPV screening should be done routinely to HIV-infected patients to increase the chances of early diagnosis and treatment.
